# P-2033. Patient Characteristics and Management of COVID-19 Diagnosed in the Outpatient Setting

**DOI:** 10.1093/ofid/ofae631.2189

**Published:** 2025-01-29

**Authors:** Mark Berry, Christian B Ramers, Manu Jain, Amos Lichtman, Ching-Yi Chuo, Anand Chokkalingam, Essy Mozaffari

**Affiliations:** Gilead Sciences, Inc., Foster City, California; Family Health Centers of San Diego, San Diego, CA; Northwestern University Feinberg School of Medicine, Chicago, Illinois; Gilead Sciences, Inc., Foster City, California; Gilead Sciences, Inc., Foster City, California; Gilead, Foster City, California; Gilead Sciences, Foster, California

## Abstract

**Background:**

The clinical profile of outpatient COVID-19 continues to evolve. Our research objective was to characterize patients diagnosed with COVID-19 in the outpatient setting and describe antiviral treatment patterns.

**Figure:**
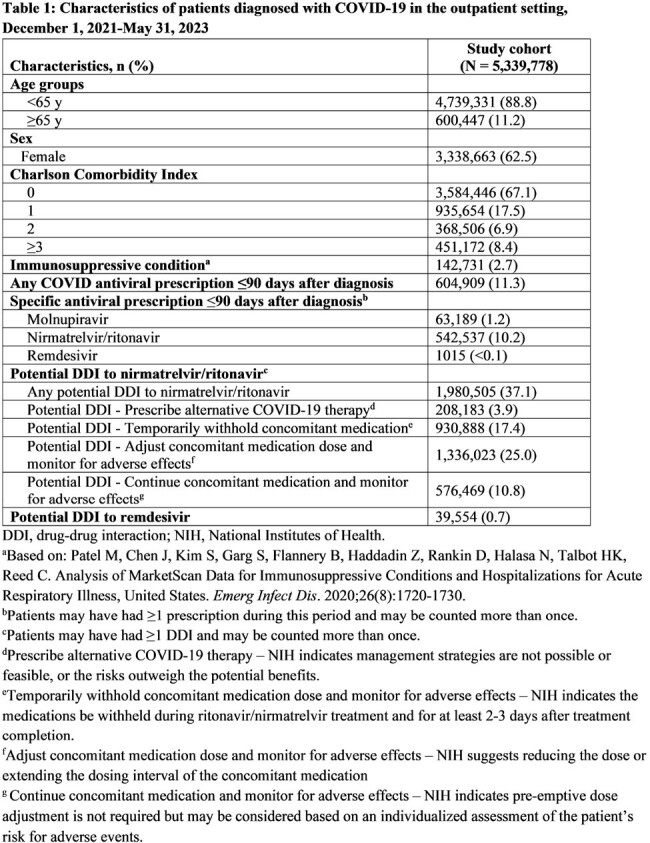

**Methods:**

The HealthVerity database (includes chargemaster, electronic health record, and claims data) was queried for individuals age ≥12 y with an outpatient COVID-19 diagnosis between 12/1/2021 and 5/31/2023. We examined patient characteristics, including demographics, Charlson Comorbidity Index (CCI), and medication prescription history. Any hospitalization ≤30 days postdiagnosis and potential drug-drug interactions (DDIs) 90 days prior to diagnosis were captured. Antiviral therapy ≤90 days postdiagnosis was also captured, though antivirals were only available under emergency use authorization during the study period.

**Figure:**
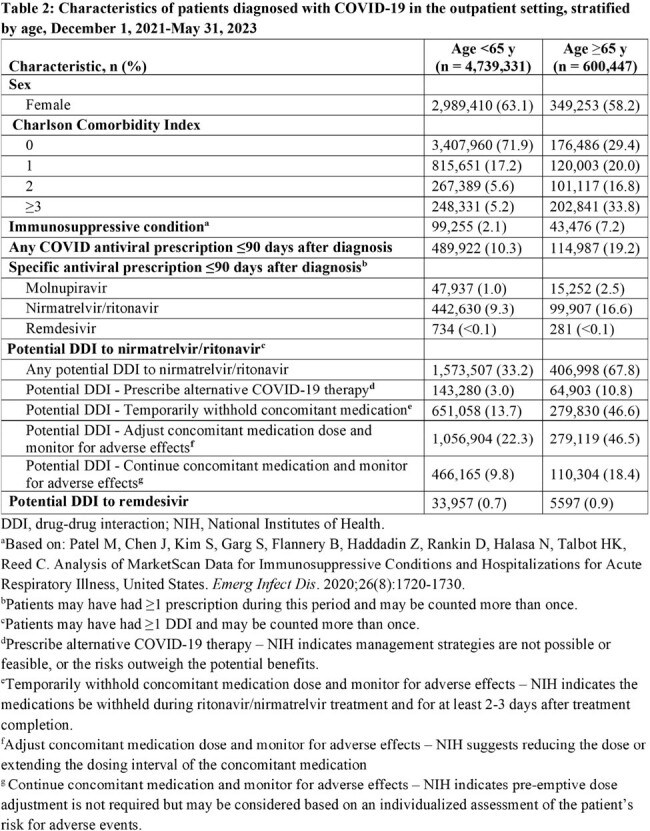

**Results:**

Over the study period, 5,339,778 patients met the entry criteria, of whom 62.5% were women and 11.2% were age ≥65 y. In addition, 2.7% had an immunosuppressive condition, 11.3% were prescribed an antiviral (10.4% molnupiravir, 89.9% nirmatrelvir/ritonavir), and 37.1% had a potential DDI to nirmatrelvir/ritonavir (Table 1). Among patients with a CCI score ≥3, 13.8% received antiviral treatment compared to 11.1% of patients with a CCI score < 3. Of patients age ≥65 y, 19.2% received antivirals compared to 10.3% of those age < 65 y. Prevalence of potential DDIs was high in patients age ≥65 y and in those with immunosuppressive conditions (Tables 2 and 3). Overall, 1.0% of patients treated for COVID-19 were hospitalized ≤30 days postdiagnosis, compared to 1.9% of those untreated (Table 4).

**Figure:**
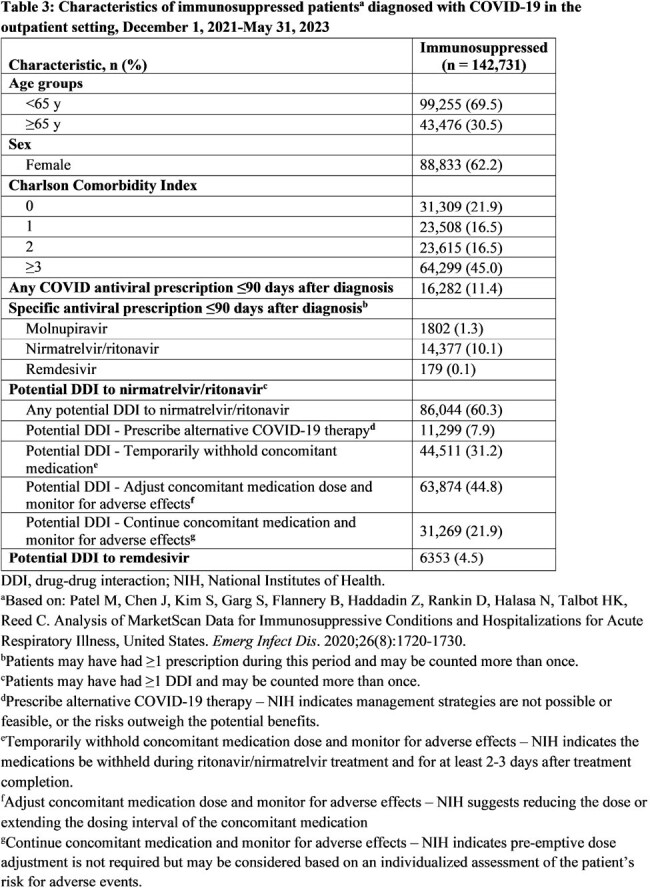

**Conclusion:**

Outpatient antiviral therapy for COVID-19 was underutilized, though there was a considerable percent of patients without comorbidities whom clinicians may have considered low-risk for serious disease progression. A high proportion of patients age ≥65 y and/or with immunosuppressive conditions were prescribed antivirals and were more likely to have potential associated DDIs. The proportion of patients who were hospitalized approximately doubled when the patient was not treated, though future adjustments will be needed to allow for subgroup comparisons, including matching of treated and untreated patients according to confounding effects.

**Figure:**
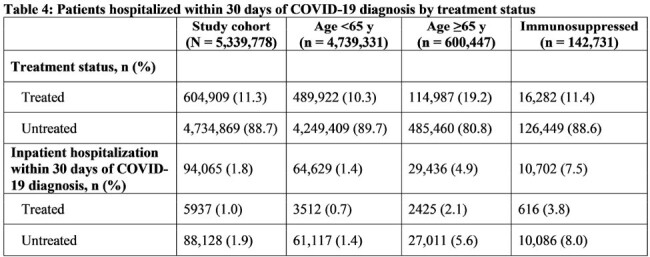

**Disclosures:**

Mark Berry, PhD, Gilead Sciences, Inc.: Employee|Gilead Sciences, Inc.: Stocks/Bonds (Public Company) Christian B. Ramers, MD MPH, AbbVie: Advisor/Consultant|AbbVie: Honoraria|Gilead Sciences, Inc.: Advisor/Consultant|Gilead Sciences, Inc.: Honoraria|Pfizer: Advisor/Consultant|Pfizer: Honoraria|Viiv: Advisor/Consultant|Viiv: Honoraria Amos Lichtman, MPH, MD, Gilead Sciences, Inc.: Employee|Gilead Sciences, Inc.: Stocks/Bonds (Public Company) Ching-Yi Chuo, PhD, Gilead Sciences, Inc.: Employee|Gilead Sciences, Inc.: Stocks/Bonds (Public Company) Anand Chokkalingam, PhD, Gilead Sciences, Inc.: Employee|Gilead Sciences, Inc.: Stocks/Bonds (Public Company) Essy Mozaffari, PharmD, MPH, MBA, Gilead Sciences, Inc.: Employee|Gilead Sciences, Inc.: Stocks/Bonds (Public Company)

